# Adipose tissue IL-23 is associated with fasting blood glucose and HbA1c in overweight/obese individuals

**DOI:** 10.3389/fendo.2025.1608846

**Published:** 2025-10-13

**Authors:** Shihab Kochumon, Fatemah Bahman, Shaima Albeloushi, Ashraf Al Madhoun, Fawaz Alzaid, Jaakko Tuomilehto, Fahd Al-Mulla, Rasheed Ahmad

**Affiliations:** ^1^ Department of Immunology and Microbiology, Dasman Diabetes Institute, Kuwait City, Kuwait; ^2^ Translational Research Department, Dasman Diabetes Institute, Kuwait City, Kuwait; ^3^ Department of Bioenergetics and Neurometabolism, Dasman Diabetes Institute, Kuwait City, Kuwait; ^4^ Institut Necker Enfants Malades (INEM), French Institute of Health and Medic Research (INSERM), Immunity and Metabolism of Diabetes (IMMEDIAB), Université de Paris Cité, Paris, France; ^5^ Department of Public Health and Welfare, Finnish Institute for Health and Welfare, Helsinki, Finland; ^6^ Department of Public Health, University of Helsinki, Helsinki, Finland

**Keywords:** adipose tissue IL-23, FBG, HbA1c, HOMA-IR, inflammatory markers, obesity

## Abstract

IL-23, a proinflammatory cytokine, plays a role in the development of inflammatory diseases. However, the association between IL-23 expression in adipose tissue (AT) and glycemic changes in obesity remains unclear. This cross-sectional study aimed to examine the relationship of adipose tissue IL-23 (AT-IL-23) expression with other inflammatory mediators within the same compartment and insulin resistance markers fasting blood glucose (FBG) and glycated hemoglobin (HbA1c) in overweight/obese individuals. Fat biopsies were collected from 10 individuals with a body mass index (BMI) < 25kg/m2 and 51 individuals with a BMI > 25kg/m2 and were analyzed for IL-23 and other inflammatory markers using qRT-PCR. Inflammatory markers, including CD16, CD11c, CCR2, CCR5, TNF-α, IL-1β, IL-2, IL-12, CCL8, CCL19, and CXCL9, were positively correlated with IL-23 in the AT-compartment in the obesity context. Notably, AT-IL-23 was correlated positively with FBG, HbA1c, and homeostasis model assessment of insulin resistance (HOMA-IR), while negatively correlating with adiponectin. These findings suggest that AT-IL-23 is associated with metabolic inflammation and insulin resistance in obesity, suggesting it may be of interest for future biomarker studies.

## 1 Introduction

Interleukin-23 (IL-23) is a heterodimeric cytokine with pro-inflammatory properties, crucial in the development and progression of several inflammatory diseases. The cytokine is predominantly secreted by activated immune cells such as monocytes, macrophages, and dendritic cells, which are present in peripheral tissues like the skin, the lining of the intestines, and the lungs (1). IL-23 can also be released by various other cells, including innate lymphoid cells, γδ T lymphocytes, and B cells (2, 3). IL-23 is composed of two subunits, p19 and p40, that work together to promote the development and proliferation of a type of T-helper cell known as Th17 cells.

(4). Th17 cells then generate inflammatory cytokines, contributing to chronic inflammation and tissue damage (5).

Elevated levels of IL-23 have been implicated in a range of autoimmune and inflammatory conditions, including colitis, gastritis, psoriasis, and arthritis (4, 6–8). Patients with rheumatoid arthritis exhibit elevated IL-23 levels in their plasma, synovial fluid, and synovial tissue within the affected joints (9, 10). Extensive evidence indicates that IL-23 is involved in regulating mucosal inflammatory responses in the gut and plays crucial roles in the pathophysiology of several diseases, including inflammatory bowel disease, ulcerative colitis, and Crohn’s disease (11–14).

IL-23, along with IL-12, functions as a critical mediator at the interface between innate and adaptive immunity by modulating antigen-presenting cells and promoting proinflammatory T cell responses (15, 16). Recent studies have shown that elevated LDL cholesterol is associated with increased IL-23 expression in adipose tissue, along with markers of inflammation, suggesting a role for IL-23 in bridging metabolic dysregulation and immune activation (17). By promoting sustained inflammation and perpetuating the immune response, IL-23 not only exacerbates several diseases, including inflammatory bowel disease, ulcerative colitis, and Crohn’s disease but also represents a promising target for therapeutic intervention aimed at modulating the immune system and alleviating autoimmune inflammatory disease symptoms (18).

Although IL-23 is recognized for its role in the progression of inflammatory diseases, the relationship between IL-23 expression in adipose tissue and glycemic changes in the context of overweight and obesity remains poorly studied. This cross-sectional study sought to explore whether IL-23 levels in adipose tissue correlate with other inflammatory mediators in the same compartment and indicators of insulin resistance, including FBG, HbA1c, and HOMA-IR, in overweight/obese individuals.

## 2 Materials and methods

### 2.1 Study population and anthropometric measurements

A total of 61 individuals without diabetes (10 individuals with BMI< 25 kg/m_2_ and 51 with BMI > 25 kg/m_2_) were recruited in this study. Among the study participants, 3 males and 2 females are taking medications (Bisoprolol, and Methyldopa) for hypertension. Among these, only two participants were using Atorvastatin, a lipid-lowering agent. All participants gave written informed consent, and the study was approved (RA# 2010–003; June 2010) by the Dasman Diabetes Institute ethics committee, which follows the updated guidelines and ethical principles for medical research involving human subjects as per World Medical Association (WMA) declaration of Helsinki. Anthropometric - measurements were determined as described earlier (19). The characteristics of the participants are summarized in **Supplementary Table S1**.

**Power Analysis – Pearson Correlation**

*A priori* power analyses for Pearson correlation were conducted using Fisher’s z-transformation and the normal approximation with bias adjustment, as implemented in SPSS. With a sample size of 61, the analysis achieved 80.1% power to detect a moderate correlation (r = 0.35), using a two-sided test at a significance level of 0.05. Likewise, with 46 participants, the analysis yielded 80.2% power to detect a correlation of r = 0.40. These power estimates demonstrate that the study is adequately powered to detect moderate-to-large effects in the relationships analyzed.

**Table:** 

Assumed Effect Size (*r*)	Sample Size (*N*)	Significance Level (α)	Achieved Power (1–β)	Test Type
0.35	61	0.05	0.801	Two-sided Pearson correlation
0.4	46	0.05	0.802	Two-sided Pearson correlation

### 2.2 Collection of subcutaneous adipose tissue

Human adipose tissue samples were obtained through a biopsy of the abdominal subcutaneous fat pad located lateral to the umbilicus, and total RNA were isolated as described previously (19).

### 2.3 Anthropometric and metabolic measurements

Peripheral blood samples were collected from individuals who had fasted overnight and were analyzed for various metabolic parameters, including FBG, lipid profile, HbA1c, fasting insulin, and adiponectin levels. The glucose and lipid profiles, including plasma triglycerides (TG), high-density lipoprotein cholesterol (HDL-C), and total cholesterol (TC), were assessed using the Siemens Dimension RXL chemistry analyzer (Diamond Diagnostics, Holliston, MA). HbA1c was quantified with the Variant device (Bio-Rad, Hercules, CA, USA). Insulin resistance was evaluated using the HOMA-IR, calculated with the formula: HOMA-IR = (fasting insulin [μU/L] × fasting glucose [nmol/L])/22.5. In our study, serum adiponectin levels were measured using the Human Adiponectin/Acrp30 Magnetic Luminex^®^ Performance Assay (Catalog #: LOBM1065, R&D Systems, Austin, TX, USA). This method was selected due to its high sensitivity (6.4 pg/mL) All assays were conducted following the manufacturer’s instructions.

### 2.4 Real‐time RT‐PCR

Total cellular RNA from adipose tissue (80 mg) was purified using RNeasy lipid tissue kit (Qiagen, Valencia, CA., USA) and following the manufacturer’s instructions. RNA samples were reverse transcribed into cDNA as instructed (High-Capacity cDNA Reverse Transcription kit; Applied Biosystems, CA, USA). To perform real‐time RT‐PCR, cDNA samples (50 ng each) were amplified (40 cycles) using TaqMan Gene Expression Master Mix (Applied Biosystems, CA, USA) and gene‐specific 20× TaqMan gene expression assays (Applied Biosystems, CA, USA) containing forward and reverse primers (**Supplementary Table S2**) and target‐specific TaqMan MGB probe labelled with FAM dye at the 5′ end and NFQ‐MGB at the 3′ end of the probe using 7500 Fast Real‐Time PCR System (Applied Biosystems, CA, USA). Each cycle involved denaturation (15 seconds at 95 °C), annealing/extension (1 minute at 60 °C) after uracil DNA glycosylases (UDG) activation (2 minutes at 50 °C) and AmpliTaq gold enzyme (10 minutes at 95 °C) activation. The amplified GAPDH expression was used as an internal control to normalize individual sample differences. The expression level of each gene target relative to the control (lean adipose tissue) was calculated using the 2−ΔΔCt method, and the relative mRNA expression was expressed as fold expression over the average control gene expression.

### 2.5 Statistical analysis

Statistical analysis was performed using GraphPad Prism software (La Jolla, CA, USA) and SPSS for Windows version 19.01 (IBM SPSS Inc., USA). Data are shown as mean ± standard deviation values, unless otherwise indicated. Unpaired Student *t*‐test was used to compare means between groups. Pearson Correlation analysis was performed to determine association between different variables. For all analyses, *P* value <0.05 was considered significant.

#### 2.5.1 Multiple linear regression

To evaluate the independent contribution of associated markers to IL-23 expression, a multiple linear regression model was conducted using SPSS. IL-23 was entered as the dependent variable, while other independent markers were included as predictors of primary interest. Age, BMI, and Sex were included as covariates to adjust for potential confounding.

Standardized β coefficients, significance values, and Variance Inflation Factors (VIFs) were reported to assess both effect sizes and multicollinearity. The significance threshold was set at α = 0.05. Model assumptions (normality, linearity, homoscedasticity) were verified through residual diagnostics.

## 3 Results

### 3.1 IL-23 gene expression in the adipose tissue is associated with inflammatory markers in overweight/obese individuals

We asked if IL-23 gene expression in the adipose tissue was associated with inflammatory markers in the same compartment. In this regard, as shown in **Table 1**, IL-23 positively correlated with M1 macrophage markers CD11c (r= 0.45, P = 0.001), CD16 (r= 0.45, P = 0.001), CD127 (r= 0.38, P = 0.01), and chemokine receptors CCR2 (r= 0.42, P = 0.004) and CCR5 (r= 0.359, P = 0.011) Interestingly, IL-23 expression in adipose tissue also positively correlated with the M2 macrophage marker CLEC7A (r = 0.641, p < 0.0001). In addition, IL-23 demonstrated a positive association with several proinflammatory cytokines, including TNF-α (r = 0.31, p = 0.031), IL-1β (r = 0.32, p = 0.046), IL-2 (r = 0.40, p = 0.005), and IL-12 (r = 0.37, p = 0.018) and proinflammatory chemokines such as CCL5 (r = 0.64, p < 0.0001), CCL19 (r = 0.33, p = 0.025), and CXCL9 (r = 0.35, p = 0.01) (**Table 1**). It is noteworthy that these outcomes were exclusively observed in overweight/obese individuals, while no significant results were detected in the lean group.

**Table 1:** IL-23 gene expression in the adipose tissue correlates with inflammatory markers.

	BMI<25 kg/m2		BMI>25 kg/m2	
	r	p	r	p
M1/M2 macrophage markers and Chemokine receptors				
CD11c	0.683	0.062	0.454	0.001**
CD16	0.489	0.181	0.453	0.001**
CD68	0.463	0.209	0.185	0.208
CD86	0.789	0.0115	0.242	0.104
CD127	0.776	0.0237	0.377	0.012*
CD141	0.042	0.929	0.325	0.023*
CD163	-0.372	0.289	0.303	0.037*
CD302	0.082	0.821	0.112	0.439
CCR1	0.501	0.14	0.042	0.779
CCR2	-0.673	0.143	0.421	0.004**
CCR5	-0.014	0.969	0.359	0.011*
CLEC7A	0.871	0.001	0.641	<0.0001****
Cytokines and Chemokines				
TNF-α	0.009	0.987	0.312	0.031*
TGF-β	0.557	0.151	0.136	0.362
IL-1β	0.858	0.142	0.318	0.046*
IL-2	0.212	0.557	0.398	0.005**
IL-6	-0.267	0.609	0.029	0.844
IL-8	0.594	0.214	0.231	0.123
IL-10	0.446	0.229	-0.063	0.674
IL-13	0.293	0.481	0.159	0.297
IL-12	0.416	0.584	0.372	0.018*
IL-18	0.577	0.104	0.148	0.325
IL-33	-0.44	0.203	-0.0003	0.998
CCL2	0.477	0.232	0.071	0.623
CCL5	-0.0001	0.999	0.636	<0.0001****
CCL8	0.206	0.624	0.083	0.6
CCL11	-0.139	0.721	0.095	0.531
CCL19	-0.224	0.533	0.331	0.025*
CCL20	0.201	0.605	0.203	0.162
CXCL9	-0.655	0.04	0.353	0.013*
CXCL10	-0.084	0.831	0.185	0.208
CXCL11	-0.537	0.109	0.199	0.175

* P<0.05.** P<0.01.**** P<0.0001.

Additionally, a multiple linear regression was conducted to assess the effect of associated markers on IL-23 expression, adjusting for age, BMI, and sex and we found CCL5 and CXCL9 as independent predictors of IL-23 (**Table 2**).

**Table 2:** Multiple linear regression results for IL-23 gene expression.

Model Fit Statistics		p-value	R²	Adjusted R²
		<.001	0.544	0.481
	Standardized Coefficients	p-value	Collinearity Statistics	
Predictors	β		Tolerance	VIF
CCL5	0.652	<0.001	0.975	1.026
CXCL9	0.358	0.004	0.951	1.051
Age	-0.095	0.415	0.959	1.042
BMI	0.116	0.316	0.971	1.030
Sex	-0.045	0.700	0.957	1.045

Multicollinearity Diagnostics

- VIFs for all predictors ranged from 1.026 to 1.051.
- Tolerance values > 0.95 for all variables.

These results confirm the independent predictive value of CCL5 and CXCL9 on IL-23 gene expression after adjusting for demographic and physiological covariates (**Table 2**). Additionally, collinearity diagnostics showed no substantial overlap in variance proportions, even at higher condition indices.

### 3.2 IL-23 gene expression in adipose tissue is related to both fasting blood glucose and glycated hemoglobin in overweight/obese individuals

**Figure 1:**
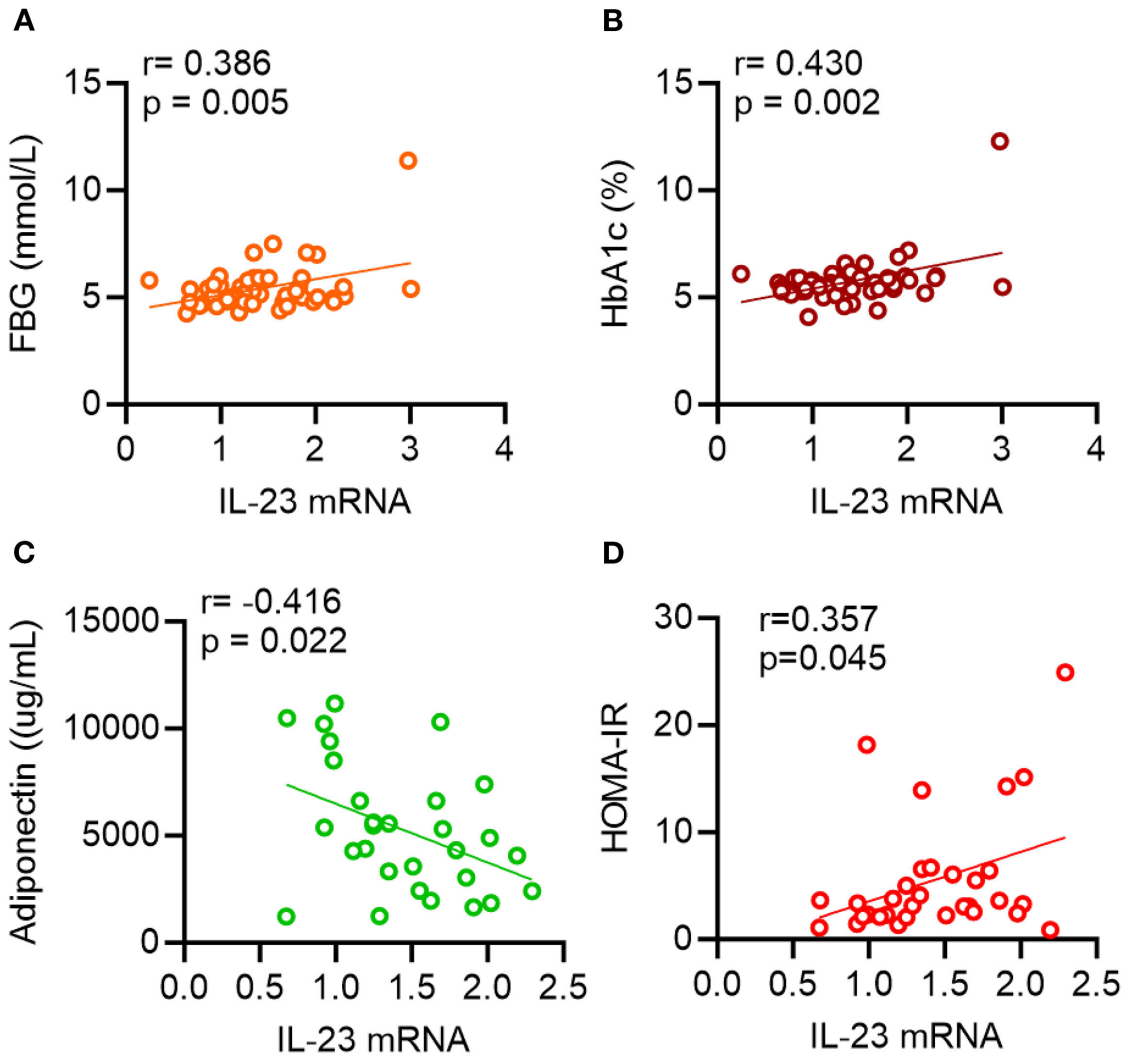
Scatterplots showing correlations between IL-23 and metabolic parameters. AT IL-23 mRNA levels were correlated with **(A)** fasting blood glucose (FBG), **(B)** glycated hemoglobin (HbA1c), **(C)** adiponectin and **(D)** HOMA-IR. Each data point represents each individual. Correlation coefficients (r) and p values were determined by Pearson’s correlation analysis.

We explored whether alterations in IL-23 gene expression within adipose tissue are linked to clinical and metabolic indicators. To this end, we measured serum levels of TG, TC, HDL-C, LDL-C, FBG, HbA1c, insulin, and adiponectin. As shown in **Table 3** and **Figure 1**, IL-23 expression was found to be positively associated with FBG (*r* = 0.39, *p* = 0.005; **Figure 1A**) and HbA1c (*r* = 0.43, *p* = 0.002; **Figure 1B**) and negatively correlated with adiponectin (*r* = −0.42, *P* = 0.02; **Figure 1C**) in overweight/obese individuals but not in the lean individuals.

**Table 3:** Correlations of adipose tissue IL-23 expression with serum metabolic markers.

	BMI<25 kg/m2		BMI>25 kg/m2	
	r	p	r	p
Chol.	-0.143	0.694	0.24	0.093
HDL	0.211	0.558	0.082	0.573
LDL	-0.297	0.405	0.22	0.125
TG	-0.025	0.946	0.038	0.795
FBG	-0.363	0.303	0.386	0.005**
HbA1c	0.342	0.334	0.43	0.002**
HOMA-IR	-0.544	0.163	0.357	0.045*
Adiponectin	0.509	0.198	-0.416	0.022*

* P<0.05.** P<0.01.

Of note, IL-23 expression in the adipose tissue of individuals with a BMI > 25 kg/m2, also positively correlated with HOMA-IR (*r* = 0.36; *P* = 0.045; **Figure 1D**), a recognized marker of insulin resistance.

## 4 Discussion

Obesity is defined as a chronic low grade inflammation which leads to metabolic dysregulation (20). The results of the present study offer important insights into the IL-23 gene expression in adipose tissue and its relationship with markers of inflammation and insulin resistance, in overweight and obese individuals. Our results show a positive association between IL-23 gene expression in adipose tissue and inflammatory markers, as well as critical metabolic indicators like FBG and HbA1c. These findings suggest a potential association between adipose tissue IL-23 expression and metabolic inflammation; however, further studies are needed to determine whether IL-23 directly contributes to metabolic dysfunction.

M1 macrophages are key contributors to inflammation within adipose tissue (21) Our findings revealed significant positive correlations between IL-23 expression and various M1 macrophage-associated markers (CD11c, CD16, CCR2, and CCR5) in the adipose tissue of overweight/obese individuals -. The observed correlations support the potential involvement of IL-23 in driving proinflammatory macrophage responses. While some functional features may overlap with the M1 phenotype, IL-23 has been shown to induce a distinct macrophage subpopulation—termed M(IL-23)—that is phenotypically and functionally different from both classical M1 and M2 macrophages (22). This unique subset is characterized by the production of IL-17A, IL-22, and IFN-γ, and contributes to Th17-cytokine-driven inflammation through pathways involving STAT3, RORγT, and T-bet. Our findings support and extend this understanding, highlighting the role of IL-23 in promoting a specialized macrophage phenotype implicated in inflammatory disease settings (22). Nevertheless, a definitive link between IL-23 levels and the expression of macrophage markers in the adipose tissue of overweight/obese individuals has not yet been established. The adipose tissue compartment is a dynamic endocrine organ that secretes a diverse range of adipocytokines (23). After identifying a correlation between adipose tissue IL-23 and elevated expression of inflammatory macrophage markers in overweight/obese individuals, we proceeded to investigate whether changes in adipose tissue IL-23 levels were linked to the local expression of inflammatory cytokines and chemokines. Our analysis revealed a positive correlation between adipose IL-23 expression and the levels of TNF-α, IL-1β, IL-12, IL-2, CCL5, CCL19, and CXCL9 in overweight/obese individuals. The role of these cytokines and chemokines in inflammation and metabolic disturbance is well documented (21, 23–25). Notably, these associations were absent in the adipose tissue of lean individuals, implying that the role of IL-23 in metabolic inflammation is likely more significant in obese individuals, where chronic low-grade inflammation is generally more common (20, 26).

These cytokines/chemokines are well-established mediators of inflammation and insulin resistance, suggesting a role for adipose tissue IL-23 in the inflammatory cascade associated with metabolic dysfunction. The positive correlations between IL-23 expression and FBG, HbA1c and HOMA-IR further support that IL-23 may be involved in regulating glucose metabolism and insulin resistance in adipose tissue. Interestingly, while our findings suggest that elevated IL-23 expression in human adipose tissue is associated with metabolic inflammation and insulin resistance, previous work in mice has reported that IL-23 knockout exacerbates obesity and glucose intolerance in the context of a high-fat diet (27). In that model, IL-23 was proposed to maintain Th17 responses and neutrophil recruitment to the gut, preventing dysbiosis and offering protection against metabolic disease. These contrasting results may reflect species-specific immune mechanisms or tissue-dependent roles of IL-23, and highlight the need for further studies to dissect IL-23’s context-dependent function in metabolic regulation, particularly in humans. Our study found a negative correlation between IL-23 expression and adiponectin levels. Adiponectin is an anti-inflammatory adipokine that plays a protective role in metabolic health by improving insulin sensitivity (28). The inverse relationship between adipose tissue IL-23 and adiponectin suggests that IL-23 may contribute to insulin resistance, which is often associated with a decrease in adiponectin levels. It is important to note that there was no association between IL-23 expression and BMI, suggesting that the relationship between adipose tissue IL-23 and metabolic markers may be more closely tied to metabolic dysfunction rather than simply adiposity itself.

In conclusion, our research emphasizes the crucial role of IL-23 in promoting inflammatory and metabolic dysfunction within the adipose tissue of overweight/obese individuals. The observed positive correlations between IL-23 and inflammatory markers (CD16, CD11c, CCR2, CCR5, TNF-α, IL-1β, IL-2, IL-12, CCL8, CCL19, CXCL9), as well as metabolic markers (FBG and HbA1c), alongside its negative association with adiponectin, highlight its potential involvement in the pathogenesis of obesity-related inflammation and insulin resistance. These findings support the notion that targeting IL-23 in adipose tissue could present a therapeutic opportunity for tackling the persistent inflammation and metabolic disorders characteristic of obesity. Nonetheless, further research is necessary to fully understand the mechanisms through which adipose tissue IL-23 contributes to these conditions and to assess its viability as a therapeutic target in obesity and metabolic disorders.

## 5 Limitations of the study

IL-23 protein levels could not be measured due to the limited quantity of adipose tissue available. In the same way, at the time of the analysis, serum samples of study subjects were no longer available for dosing serum IL-23. In addition, although IL-23 is known to be produced by tissue-resident myeloid cells, we did not directly determine its cellular source in adipose tissue. Future studies will be necessary to confirm these findings at the protein level and to characterize the specific cell types responsible for IL-23 production in obesity.

The second limitation of our study is the unequal group sizes, with fewer lean adipose tissue samples available (n=10) compared to the obese group (n=51). This discrepancy reflects the difficulty of obtaining adipose biopsies from lean individuals, which are less frequently available for research purposes. While this smaller sample size may limit the statistical power to detect associations within the lean group, it does not diminish the robustness of the findings observed in the obese group, where the sample size was larger and correlations with IL-23 expression were consistent and biologically meaningful.

## Data Availability

The original contributions presented in the study are included in the article/**Supplementary Material**. Further inquiries can be directed to the corresponding author.

## Supplementary material

Supplementary Table 1 Anthropometric, biochemical, and clinical characteristics of study participants.

Supplementary Table 2 Primer Assay ID’s.
