# Supplementary material for: Adipose tissue IL-23 is associated with fasting blood glucose and HbA1c in overweight/obese individuals
Source: Front Endocrinol (Lausanne). 2025 Oct 13;16:1608846. doi: 10.3389/fendo.2025.1608846 (PMC12554591; doi:10.3389/fendo.2025.1608846)
Supplement: Supplementary Table 1 — Anthropometric, biochemical, and clinical characteristics of study participants. [file DataSheet1.pdf]

Supplementary Tables

Table S1: Clinico-demographic data of study participants

Physical and Biochemical Characteristics	BMI≤25 kg/m2 (n = 10) (Mean ± SD)	BMI>25 kg/m2 (n =51) (Mean ± SD)	p value
Age (years)	42.70±8.17	44.31±12.13	0.689
Weight (kg)	62.93±11.90	88.39±14.50	<0.0001
Height (m)	1.66±0.12	1.65±0.11	0.948
BMI (kg/m2)	22.82±2.35	32.28±4.30	<0.0001
Waist circumference (cm)	81.33±12.44	102.55±12.95	<0.0001
Body fat (%)	28.37±6.27	36.92±5.73	0.0002
Fasting blood glucose (mmol/L)	4.97±0.64	5.43±1.11	0.208
Triglycerides (mmol/L)	0.63±0.24	1.28±0.76	0.010
Total cholesterol (mmol/L)	5.30±1.11	4.97±0.95	0.324
HDL cholesterol (mmol/L)	1.69±0.51	1.22±0.30	0.0002
LDL (mmol/L)	3.31±0.93	3.19±0.84	0.676
HbA1c (%)	5.66±0.46	5.75±1.10	0.793

Table S2: Primer Assay ID's.

Gene	Assay ID	Gene	Assay ID
IL-1β	Hs01555410_m1	CCL-19	Hs00171149_m1
IL-2	Hs00174114_m1	CCL-20	Hs01011368_m1
IL-6	Hs00985639_m1	CXCL9	Hs00171065_m1
IL-8	Hs00174103_m1	CXCL10	Hs01124251_g1
IL-10	Hs00961622_m1	CXCL-11	Hs04187682_g1
IL-12A	Hs01073447_m1	CCR1	Hs00928897_s1
IL-13	Hs00174379_m1	CCR2	Hs00704702_s1
IL-18	Hs01038788_m1	CCR5	Hs99999149_s1
IL-23A	Hs00900828_g1	CD11c	Hs00174217_m1
IL-33	Hs00369211_m1	CD16	Hs04334165_m1
TNF-α	Hs01113624_g1	CD68	Hs02836816_g1
TGF-β	Hs00820148_g1	CD86	Hs01567026_m1
CLEC7A	Hs01902549_s1	CD127	Hs00902334_m1
CCL2	Hs00234140_m1	CD141	Hs00264920_s1
CCL5	Hs00982282_m1	CD163	Hs00174705_m1
CCL8	Hs04187715_m1	CD302	Hs00994886_m1
CCL-11	Hs00237013_m1	GAPDH	Hs03929097_g1
